# Supplementary material for: An automated workflow for multi-omics screening of microbial model organisms
Source: NPJ Syst Biol Appl. 2023 May 19;9:14. doi: 10.1038/s41540-023-00277-6 (PMC10199033; doi:10.1038/s41540-023-00277-6)
Supplement: Supplementary file 4 — Reporting Summary [file 41540_2023_277_MOESM4_ESM.pdf]

nature portfolio

Corresponding author(s): Lars Keld Nielsen, Douglas McCloskey

Last updated by author(s): Mar 6, 2023

Reporting Summary

Nature Portfolio wishes to improve the reproducibility of the work that we publish. This form provides structure for consistency and transparency in reporting. For further information on Nature Portfolio policies, see our Editorial Policies and the Editorial Policy Checklist.

Statistics

For all statistical analyses, confirm that the following items are present in the figure legend, table legend, main text, or Methods section.

n/a	Confirmed	
☐	☒	The exact sample size (*n*) for each experimental group/condition, given as a discrete number and unit of measurement
☐	☒	A statement on whether measurements were taken from distinct samples or whether the same sample was measured repeatedly
☒	☐	The statistical test(s) used AND whether they are one- or two-sided<br>*Only common tests should be described solely by name; describe more complex techniques in the Methods section.*
☒	☐	A description of all covariates tested
☒	☐	A description of any assumptions or corrections, such as tests of normality and adjustment for multiple comparisons
☐	☒	A full description of the statistical parameters including central tendency (e.g. means) or other basic estimates (e.g. regression coefficient) AND variation (e.g. standard deviation) or associated estimates of uncertainty (e.g. confidence intervals)
☒	☐	For null hypothesis testing, the test statistic (e.g. *F*, *t*, *r*) with confidence intervals, effect sizes, degrees of freedom and *P* value noted<br>*Give P values as exact values whenever suitable.*
☒	☐	For Bayesian analysis, information on the choice of priors and Markov chain Monte Carlo settings
☒	☐	For hierarchical and complex designs, identification of the appropriate level for tests and full reporting of outcomes
☒	☐	Estimates of effect sizes (e.g. Cohen's *d*, Pearson's *r*), indicating how they were calculated

Our web collection on statistics for biologists contains articles on many of the points above.

Software and code

Policy information about availability of computer code

Data collection	i-control v 1.12 was used to acquire data on the TECAN Nano Plus plate reader. Tecan EvoWare software v 8.0 with integrated Visual Basic modules were used to control the custom TECAN cultivation platform. Custom lids were designed with SolidWorks CAD. Metabolomics data was acquired using Sciex Analyst version 1.7. HPLC analysis was conducted using Chromeleon v 7.2.9. Proteomics data was acquired using Xcalibur v 4.4. Amino acid isotopomer analysis was conducted using MassHunter v 10.0.
Data analysis	A custom Python-based module was developed to process the acquired OD measurements, available at https://pypi.org/project/tecan-od-analyzer. Proteomics data was processed using Spectronaut 15.4. Metabolomics and fluxomics data was processed using ProteoWizard v 3.0 and SmartPeaks v 1.2, further analysis was performed with custom Matlab scripts and INCA v 2.0. Phenomics data was processed using SmartPeaks v 1.2.

For manuscripts utilizing custom algorithms or software that are central to the research but not yet described in published literature, software must be made available to editors and reviewers. We strongly encourage code deposition in a community repository (e.g. GitHub). See the Nature Portfolio guidelines for submitting code & software for further information.

Data

Policy information about availability of data

All manuscripts must include a data availability statement. This statement should provide the following information, where applicable:

- Accession codes, unique identifiers, or web links for publicly available datasets
- A description of any restrictions on data availability
- For clinical datasets or third party data, please ensure that the statement adheres to our policy

Processed data for growth, uptake/secretion rates, metabolomics and proteomics is available respectively in Supplementary Tables 1-4. Raw data is available from the authors upon request.

Human research participants

Policy information about studies involving human research participants and Sex and Gender in Research.

Reporting on sex and gender	not applicable
Population characteristics	not applicable
Recruitment	not applicable
Ethics oversight	not applicable

Note that full information on the approval of the study protocol must also be provided in the manuscript.

Field-specific reporting

Please select the one below that is the best fit for your research. If you are not sure, read the appropriate sections before making your selection.

☒ Life sciences ☐ Behavioural & social sciences ☐ Ecological, evolutionary & environmental sciences

For a reference copy of the document with all sections, see nature.com/documents/nr-reporting-summary-flat.pdf

Life sciences study design

All studies must disclose on these points even when the disclosure is negative.

Sample size	Experiments in micro-titer plates were conducted to maximize the amount of samples considering blank samples and concentration (n = 80). Other experiments were carried out in triplicates.
Data exclusions	Identification of outlier samples is detailed in Supplementary Materials.
Replication	All attempts at replication were succesfull.
Randomization	Not relevant, we used replicates of the same strains in the same conditions.
Blinding	Not relevant, we used replicates of the same strains in the same conditions.

Reporting for specific materials, systems and methods

We require information from authors about some types of materials, experimental systems and methods used in many studies. Here, indicate whether each material, system or method listed is relevant to your study. If you are not sure if a list item applies to your research, read the appropriate section before selecting a response.

Materials & experimental systems

n/a	Involved in the study
☒	☐ Antibodies
☒	☐ Eukaryotic cell lines
☒	☐ Palaeontology and archaeology
☒	☐ Animals and other organisms
☒	☐ Clinical data
☒	☐ Dual use research of concern

Methods

n/a	Involved in the study
☒	☐ ChIP-seq
☒	☐ Flow cytometry
☒	☐ MRI-based neuroimaging
